# Inverse association between blood ethylene oxide levels and obesity in the general population: NHANES 2013–2016

**DOI:** 10.3389/fendo.2022.926971

**Published:** 2022-09-12

**Authors:** Iokfai Cheang, Xu Zhu, Qingqing Zhu, Menghuan Li, Shengen Liao, Zhi Zuo, Wenming Yao, Yanli Zhou, Haifeng Zhang, Xinli Li

**Affiliations:** ^1^ Department of Cardiology, The First Affiliated Hospital of Nanjing Medical University, Jiangsu Province Hospital, Nanjing, China; ^2^ Department of Cardiology, Affiliated Suzhou Hospital of Nanjing Medical University, Suzhou Municipal Hospital, Suzhou, China

**Keywords:** ethylene oxide (EO), obesity, abdominal obesity, inflammation, NHANES, epidemiology

## Abstract

**Background:**

Ethylene oxide (EO) has been shown to associate with increased cardiovascular risk. This study aimed to explore the relationship and its meditating factors between EO exposure and the major cardiovascular risk factor of obesity among the general adult population.

**Methods:**

Cross-sectional data of 3,220 participants from National Health and Nutritional Examination Survey (NHANES) 2013–2016 were enrolled. Obesity was defined as body mass index (BMI) ≥30 kg/m^2^, and abdominal obesity was defined as waist circumference (WC) ≥102 cm in men and ≥88 cm in women. The association among hemoglobin adduct of EO (HbEO), inflammatory biomarkers, and obesity was evaluated using restricted cubic splines and the multivariable linear regression model. Mediation analysis was used to further assess their association.

**Results:**

The increased quartiles of HbEO were inversely associated with BMI and WC [Q1 vs. Q4, BMI: *β* = −2.98 (−3.74, −2.22), WC: *β* = −6.50 (−8.60, −4.39); all *p* for trend < 0.05], and were inversely associated with obesity after full adjustment [obesity: OR = 0.43 (0.31, 0.58), abdominal obesity: OR = 0.42 (0.27, 0.65); all *p* for trend < 0.05]. The levels of alkaline phosphatase, white blood cells, lymphocytes, and neutrophils were also positively associated with BMI and WC (all *p* < 0.05). Mediation analysis showed that exposure of EO not only had a negative direct effect on BMI and WC, but also generated an inverse indirect effect.

**Conclusions:**

Current findings showed an inverse association between HbEO and obesity, and suggested that systemic inflammation may not be their only mediator. Additional research is required to explore the underlying link of EO and system metabolism.

## Introduction

As a metabolite of ethylene, ethylene oxide (EO) is a ubiquitous compound in the environment (1). EO is a widely used industrial chemical for manufacturing various daily products such as antifreeze, polyester, detergents, adhesives, textiles, solvents, and pesticides. Exposure to EO in the general population may be through application of sterilized medical equipment (2), fumigation of food and cosmetics (3), and inhalation of contaminated air, tobacco smoke, and/or vehicle exhaust (4, 5). Exposure to EO raises various health issues. EO is a highly reactive organic compound causing the alkylation of DNA, RNA, and proteins (1). Previous large prospective general population studies have shown that the exposure to EO is associated with an increased risk of cancer and has been classified as a Group 1 human carcinogen by the International Agency for Research on Cancer (6). In addition, studies have also suggested that chronic EO exposure may be correlated (7, 8) with the systemic inflammatory response.

The use of EO sterilization in personal protective equipment (PPE) plays an important role in the COVID-19 pandemic. Notably, the EO residue in regulatory compliance single-use sterile medical devices, products, and PPEs (for example, the residual EO on face masks <10 μg/g) should be overall safe. However, the wide application of PPE potentially increased the exposure to EO, which leads to substantial confusion about chronic EO exposures for the general population (9, 10). Furthermore, the chronic exposure to EO is also associated with various cardiovascular risk factors such as smokers, serum lipid levels, and diabetes (1, 11–14). In our previous study, EO is associated with dyslipidemia and that might be mediated by the systemic inflammation (15). While the above evidence supports that the environmental pollutants such as EO may have an adverse effect on the cardiovascular system (16), only limited studies have explored such associations and the potential inflammatory mechanism.

Studies have also shown a significant association between exposure to a variety of environmental chemicals and obesity in humans (17). Meanwhile, the worldwide epidemic of overweight and obesity continues to be a major challenge in chronic disease prevention. Obesity leads to significant adverse health effects due to its association with metabolic disturbances and is a major cardiovascular risk factor (18, 19). Obesity increases cardiovascular risk through lipid abnormalities. The development of insulin resistance, hepatic fatty acid metabolic disturbance, intravascular lipolysis, adipose tissue dysfunctionality, and chronic inflammation is also closely related to obesity, metabolic syndrome, and dyslipidemia (20–22).

However, the relationship between EO and obesity remained unclear. Herein, our study hypothesized that the serum level of EO adduct hemoglobin (HbEO) might be associated with obesity, which is mediated by inflammation. The objective of the current study is to demonstrate the association of HbEO levels with body mass parameters and obesity, and the mediating role of systemic inflammatory biomarkers in the general population.

## Methods

### Study participants

This cross-sectional epidemiologic study used publicly available data from the National Health and Nutrition Examination Survey (NHANES) conducted by the US National Center for Health Statistics (NCHS). Authors have not been involved in the collection and production of the database in use. The NHANES survey represents the noninstitutionalized civilian population in the US. All participants provided signed informed consent in accordance with the protocol approved by the ethics review board of NCHS. The detailed obtained ethics approval and written informed consent are available at https://www.cdc.gov/nchs/nhanes/irba98.htm. The survey design, methods, and data protocols are publicly available at https://www.cdc.gov/nchs/nhanes/about_nhanes.htm.

**Figure 1:**
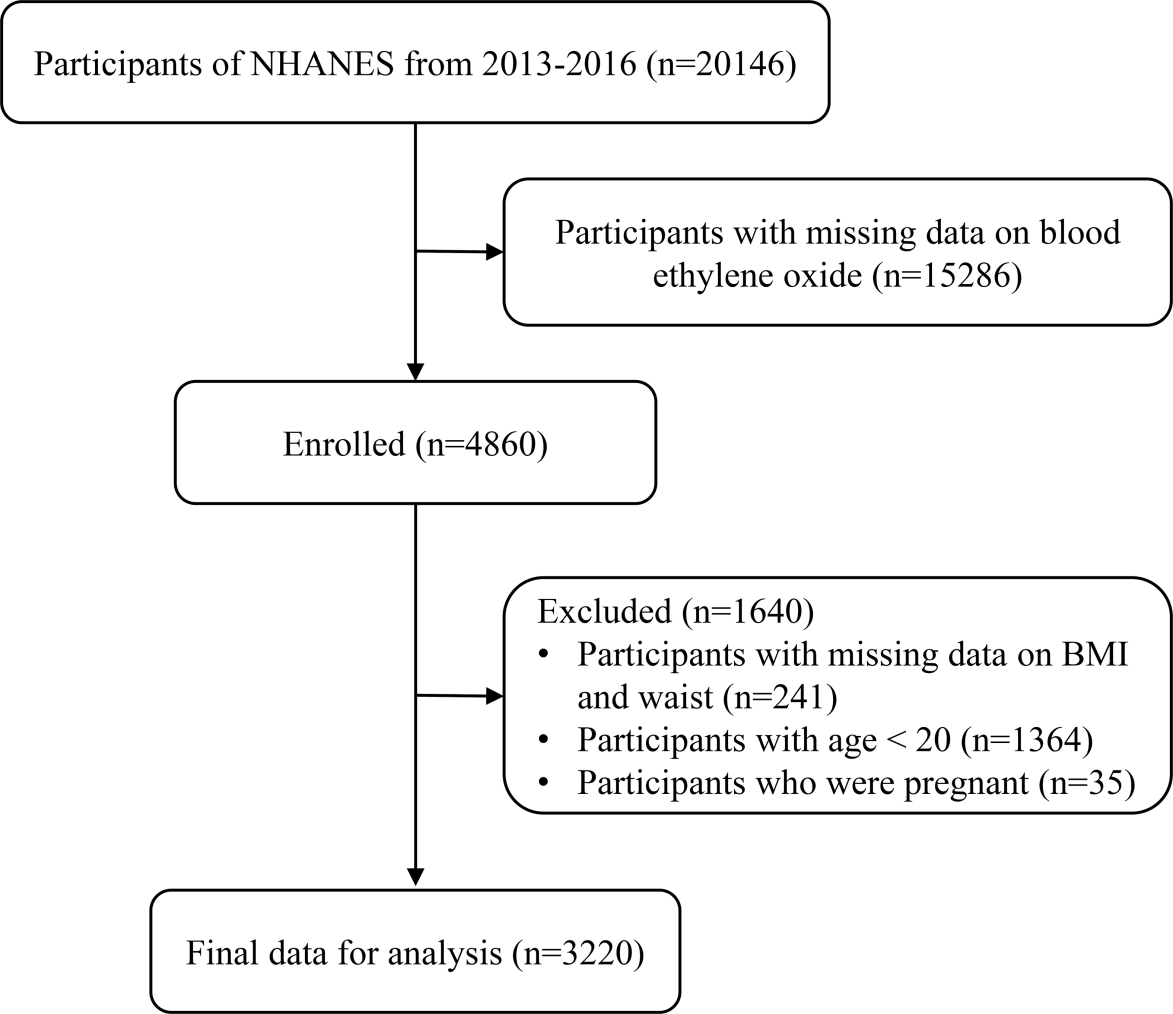
Study population flowchart. NHANES, National Health and Nutrition Examination Survey; BMI, body mass index.

Among NHANES 2013–2016, including three survey cycles in the total sample, there were 3,220 participants enrolled in the analysis. Participants without blood EO, underage participants, those with missing data on weight and waist indexes, and those who are pregnant were excluded (**Figure 1**).

### Anthropometric measures

Weight, height, and waist circumference (WC) were measured at the Mobile Examination Centers (MECs) using the standard methodology by using the standardized procedure manual. Body mass index (BMI) is calculated by dividing body weight in kilograms by height in meters squared (kg/m^2^). Obesity was defined as BMI at or above 30 kg/m^2^. While WC adds critical information along with BMI, WC was measured with a tape measure at the uppermost lateral border of the hip crest. WC ≥102 cm in men and ≥88 cm in women were considered abdominal obesity (20).

### Blood ethylene oxide levels

Hemoglobin adduct of ethylene oxide (HbEO) has been shown to be a highly sensitive method for determining exposure to EO (23).

Blood samples were obtained in the morning from participants who had fasted for at least 9 h in MECs. Washed-packed red blood cell specimens were processed and stored at −30°C until preset batches of samples were supplied to technicians for analysis on a regular basis. Human whole blood or erythrocyte levels were determined to assess HbEO. The reaction products with the N-terminal valine of the hemoglobin protein chains (N-[2-carbamoyl ethyl] valine and N-[2-hydroxycarbamoyl-ethyl] valine EO adducts) were measured using the modified Edman reaction by high-performance liquid chromatography coupled with tandem mass spectrometry (HPLC-MS/MS) with the corresponding commercial assay kit (Tech Diagnostics, Anaheim, CA). For analytes with analytical results less than the lower limit of detection (12.9 pmol/g Hb), the values were replaced with the lower limit of detection divided by the square root of two (LLOD/sqrt (2)). The method for HbEO measurement is provided at https://wwwn.cdc.gov/Nchs/Nhanes/2015-2016/ETHOX_I.htm.

### Covariates

NHANES uses a complex sampling design and constructs sample weights to produce nationally representative data. Sampling weights are used to produce correct population estimates because each sample person does not have an equal probability of selection. Survey weights are constructed for each 2-year cycle, which considers non-response, over-sampling, post-stratification, and sampling error. Details for sampling weight are found at https://wwwn.cdc.gov/nchs/nhanes/tutorials/module3.aspx.

The demographic characteristics including age, sex, educational level, race, income status, smoking status, alcohol consumption, sedentary time, daily energy intake, and past medical history (hypertension and diabetes mellitus) were obtained during the family interview using a standardized questionnaire.

Educational levels were classified into three categories: below high school, high school, and above high school. Race and ethnicity were classified as follows: Mexican American, other Hispanic, non-Hispanic white, non-Hispanic black, and other race groups (including multiracial). The family poverty income ratio (PIR) was used to assess the economic status. Poverty is characterized as a family PIR of less than one. Alcohol users were identified as individuals who consumed at least 12 alcohol drinks in a single calendar year. A smoker was defined as someone who had smoked at least 100 cigarettes throughout the course of his or her lifetime. The number of minutes of sedentary behavior on a typical day was assessed by interviewing participants. Lipid profiles (total cholesterol [TC] and high-density lipoprotein cholesterol [HDL-C]) and inflammation markers (including alkaline phosphatase [ALP], white blood cell count [WBC], neutrophil count [NEU], and lymphocyte count [LYM]) in the corresponding cycles were obtained from the laboratory data.

### Statistical analysis

Weighting was performed prior to the analysis to adjust for sample frame based on independent population. Examination sample weights, which account for the differential probabilities of selection, nonresponse, and noncoverage, were incorporated into the estimation process using the “survey” package in R studio.

To verify the normal distribution of continuous variables, a Kolmogorov–Smirnov statistical test was performed. Continuous variables are presented as the means [standard deviations (SDs)] with normal distribution or medians with interquartile ranges (IQRs, Q1–Q3) with non-normal distribution. Categorical variables are presented as numbers (%). HbEO levels were log2-transformed to normalize the distributions. To investigate the dose–response curves between HbEO levels and the anthropometric parameters, restricted cubic splines with knots were used at the 5th, 35th, 65th, and 95th percentiles of HbEO level distribution. HbEO was divided into quartiles (Q) with the lowest quartile serving as the reference group to further evaluate the relationships with inflammatory markers, and serum lipid profiles using multivariable linear regression models. Model 1 was unadjusted. Model 2 was adjusted for age, sex, education level, and race. Model 3 was the same as Model 2 with additional adjustments for poverty, smoker, alcohol user, energy intake levels, sedentary time, TC, HDL-C, diabetes, and hypertension.

To determine the extent to which the relationship between HbEO levels and anthropometric parameters was mediated by inflammation (ALP, WBC, NEY, and LYM), causal mediation analyses were applied to estimate the direct effect (DE, mediator to outcome), indirect effect (IE, exposure to mediator), and total effect (TE, exposure to outcome). The 1,000 bootstrapping was used for significance testing, and the analyses were adjusted for the same covariates in Model 3 (24, 25).

The statistical analyses were performed by SPSS (version 24.0; IBM) and R software (version 3.6.0; The R Foundation for Statistical Computing). Two-sided *p* < 0.05 was considered statistically significant.

## Results

### Baseline characteristics

**Table 1** demonstrated the characteristics of the study population. The study included 2,721 participants, 49.2% of whom are male with a median age of 47 years. For the prevalence of the obese-related lifestyle and risk factors, there were 31.2% of participants with hypertension, 10.4% with diabetes, 14.9% in poverty, 43.9% with smoking habits, 77.2% were alcohol users, and 49.3% with sedentary time >6 h. The median BMI in the overall population was 28.0 (24.5, 32.7) kg/m^2^, and the median WC was 99.0 (88.2, 109.7) cm. A total of 1,262 (38.9%) individuals were considered in the obese category, and 1,863 (58.8%) were in the abdominal obesity category. The median HbEO level in the studied population was 39.7 (21.8, 55.7) pmol/g·Hb. Median TC was 188.0 (162.0, 214.0) mg/dl and median HDL-C was 51.0 (42.0, 64.0) mg/dl.

**Table 1:** Survey-weighted, sociodemographic, and health status characteristics of adult NHANES 2013–2016 participants with available blood ethylene oxide (*n* = 2,721).

Variable	Median (IQR) or *N* (%)
Age, years	47.0 (33.0, 61.0)
Male, %	1,606 (49.2%)
Education level, %	
Below high school	697 (14.0%)
High school	721 (21.2%)
Above high school	1,802 (64.8%)
Race/ethnicity, %	
Mexican American	498 (9.0%)
Other Hispanic	366 (6.0%)
Non-Hispanic White	1,258 (65.8%)
Non-Hispanic Black	633 (10.8%)
Other races	465 (8.4%)
Poverty, %	709 (14.9%)
Smoker, %	1,395 (43.9%)
Alcohol use, %	2,281 (77.2%)
Sedentary time, h	
<3 h	330 (8.2%)
3–6 h	1,424 (42.5%)
>6 h	1,466 (49.3%)
Energy intake, kcal/day	2,046.0 (1,535.0, 2,621.0)
Hypertension, %	1,138 (31.2%)
Diabetes, %	425 (10.4%)
TC, mg/dl	188.0 (162.0, 214.0)
HDL-C, mg/dl	51.0 (42.0, 64.0)
HbEO, pmol/g Hb	39.7 (21.8, 55.7)
Body mass index, kg/m^2^	28.0 (24.5, 32.7)
Waist, cm	99.0 (88.2, 109.7)
Obesity, %	1,262 (38.9%)
Abdominal obesity, %	1,863 (58.8%)

Data are presented as median (IQR) or n (%). Sampling weights were applied for calculation of demographic descriptive statistics. IQR, interquartile range; TC, total cholesterol; HDL-C, high-density lipoprotein cholesterol; HbEO, hemoglobin adduct of ethylene oxide.

### Association between HbEO and anthropometric parameters

The relationships between the HbEO level and anthropometric parameters (BMI and WC) in both continuous and categorical analyses are presented in **Table 2**. The continuous analysis showed that log2-transformed HbEO had a significant inverse association with BMI and WC regardless of adjustment (*p* < 0.001).

**Table 2:** Multiple linear regression associations of HbEO with anthropometric parameters in adults.

Outcomes	Model	Continuous log2-transformed EO	Quartile 1	Quartile 2	Quartile 3	Quartile 4	*P* for trend
			*β*	*β* (95% CI)	*β* (95% CI)	*β* (95% CI)	
BMI	Model 1	−0.53 (−0.70, −0.36) ^***^	0.00 (Ref.)	0.11 (−0.80, 1.01)	−1.04 (−2.05, −0.03) ^*^	−1.51 (−2.17, −0.84) ^***^	<0.001
	Model 2	−0.55 (−0.71, −0.39) ^***^	0.00 (Ref.)	0.08 (−0.82, 0.98)	−1.04 (−2.09, 0.01)	−1.53 (−2.23, −0.83) ^***^	<0.001
	Model 3	−0.92 (−1.12, −0.72) ^***^	0.00 (Ref.)	−0.25 (−1.03, 0.53)	−1.35 (−2.41, −0.29) ^*^	−2.98 (−3.74, −2.22) ^***^	<0.001
WC	Model 1	−1.08 (−1.49, −0.67) ^***^	0.00 (Ref.)	0.58 (−1.40, 2.57)	−2.55 (−5.01, −0.09) ^*^	−2.87 (−4.58, −1.15) ^**^	0.004
	Model 2	−1.05 (−1.47, −0.63) ^***^	0.00 (Ref.)	0.39 (−1.61, 2.38)	−2.52 (−4.85, −0.19) ^*^	−2.61 (−4.50, −0.73) ^**^	0.018
	Model 3	−2.04 (−2.57, −1.52) ^***^	0.00 (Ref.)	−0.50 (−2.26, 1.26)	−3.40 (−5.69, −1.11) ^**^	−6.50 (−8.60, −4.39) ^***^	<0.001

Model 1 was not adjusted.

Model 2 was adjusted for age, sex, education level, and race.

Model 3 was adjusted as model 2 plus poverty, smoker, alcohol user, energy intake levels, sedentary time, total cholesterol, high-density lipoprotein cholesterol, diabetes, and hypertension.

HbEO, hemoglobin adduct of ethylene oxide; BMI, body mass index; WC, waist circumference; CI, confidence interval; Ref., reference; ^*^p < 0.05, ^**^p < 0.01, and ^***^p < 0.001.

Compared with the lowest quartile, the adjusted *β* (95% CI) in the highest quartile was −2.98 (−3.74, −2.22) for BMI and −6.50 (−8.60, −4.39) for WC after full adjustment (Model 3, both *p* < 0.001). Also regardless of the adjustment, when compared with the lowest quantiles of HbEO, it remained a significant trend in both BMI (*p* for trend <0.001) and WC (*p* for trend <0.05).

**Figure 2:**
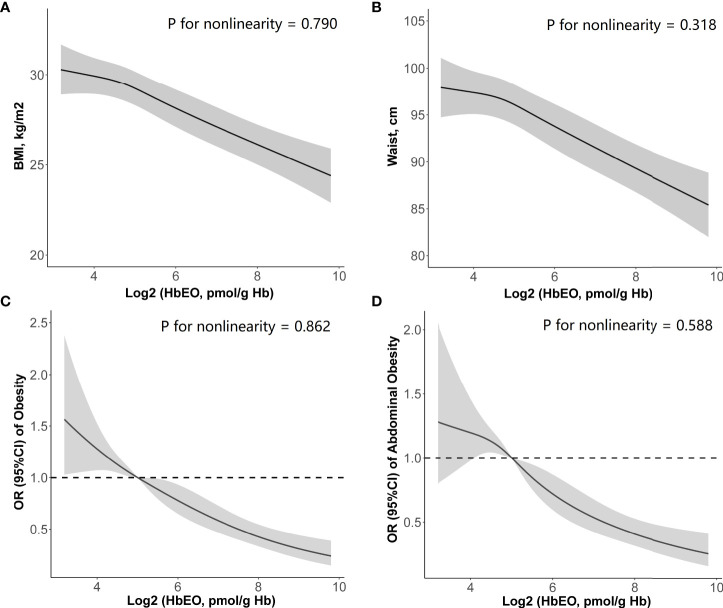
Restricted cubic spline (RCS) plots of the association of HbEO levels with **(A)** body mass index, **(B)** waist circumference, **(C)** prevalence of obesity, and **(D)** prevalence of abdominal obesity. Adjusted for age, sex, education level, race, poverty, smoker, alcohol user, energy intake levels, sedentary time, total cholesterol, high-density lipoprotein cholesterol, diabetes, and hypertension. HbEO, hemoglobin adduct of ethylene oxide; BMI, body mass index.

In addition, the cubic spline curve (including log2-transformed HbEO and four blood lipids as continuous variables) also yielded inverse trends similar to the above results (**Figure 2A**: BMI; **Figure 2B**: WC). HbEO exhibited a linear association with BMI and WC (*p* for nonlinearity >0.05).

### Association between HbEO and obesity

The associations between HbEO and the prevalence of obesity (both BMI-defined obesity and WC-defined abdominal obesity) are shown in **Table 3**. The continuous analysis showed that HbEO also had a significant inverse association with obesity regardless of adjustment (*p* < 0.01).

**Table 3:** Multiple logistic regression associations of HbEO with obesity and abdominal obesity in adults.

Outcomes	Model	Continuous log2-transformed EO	Quartile 1	Quartile 2	Quartile 3	Quartile 4	*P* for trend
			OR	OR (95% CI)	OR (95% CI)	OR (95% CI)	
Obesity	Model 1	0.89 (0.83, 0.96) ^**^	1.00 (Ref.)	0.85 (0.61, 1.19)	0.74 (0.55, 0.99) ^*^	0.71 (0.56, 0.90) ^**^	0.027
	Model 2	0.88 (0.81, 0.95) ^**^	1.00 (Ref.)	0.84 (0.60, 1.17)	0.73 (0.53, 1.00)	0.68 (0.52, 0.89) ^**^	0.027
	Model 3	0.77 (0.70, 0.85) ^***^	1.00 (Ref.)	0.71 (0.49, 1.04)	0.62 (0.44, 0.89) ^*^	0.43 (0.31, 0.58) ^***^	<0.001
Abdominal obesity	Model 1	0.88 (0.83, 0.94) ^***^	1.00 (Ref.)	0.97 (0.71, 1.33)	0.71 (0.50, 1.00) ^*^	0.69 (0.52, 0.90) ^**^	0.014
	Model 2	0.90 (0.84, 0.96) ^**^	1.00 (Ref.)	0.96 (0.68, 1.37)	0.71 (0.48, 1.07)	0.79 (0.59, 1.06)	0.198
	Model 3	0.74 (0.67, 0.82) ^***^	1.00 (Ref.)	0.84 (0.56, 1.25)	0.57 (0.37, 0.89) ^*^	0.42 (0.27, 0.65) ^***^	0.001

Model 1 was not adjusted by any covariate.

Model 2 was adjusted for age, sex, education level, and race.

Model 3 was adjusted as model 2 plus poverty, smoker, alcohol user, energy intake levels, sedentary time, total cholesterol, high-density lipoprotein cholesterol, diabetes, and hypertension.

HbEO, hemoglobin adduct of ethylene oxide; CI, confidence interval; Ref., reference; ^*^p < 0.05, ^**^p < 0.01 and ^***^p < 0.001.

After adjustment of a series of covariates, HbEO was significantly and inversely associated with obesity (*p* for trend <0.0001). Compared with the lowest quartile, the multivariate odds ratios (ORs) and 95% confident intervals (95% CIs) across the increasing quartiles of HbEO declined as 0.71 (0.49, 1.04), 0.62 (0.44, 0.89), and 0.43 (0.31, 0.58) (Model 3: *p* for trend <0.001). Similar association between HbEO and abdominal obesity was also found across the increasing quartiles compared with the reference quartile in the fully adjusted model [Model 3: OR = 0.84 (0.56, 1.25), 0.57 (0.37, 0.89), and 0.42 (0.27, 0.65); *p* for trend = 0.001]. In the restricted cubic spline curve, HbEO also had a negative correlation and linear association with obesity (*p* for nonlinearity = 0.862, **Figure 2C**) and abdominal obesity (*p* for nonlinearity = 0.588, **Figure 2D**).

### Mediation effect of systemic inflammation between HbEO and anthropometric parameters

There were significant correlations between the inflammatory markers (ALP, WBC, NEY, and LYM) and anthropometric parameters (BMI and WC) in multivariate linear regression models (all *p* < 0.01, **Table 4**). In addition, HbEO was significantly associated with inflammation markers (all *p* for trend < 0.05, **Table S1**).

**Table 4:** Multiple linear regression associations of inflammatory markers with anthropometric parameters in adults.

	BMI		WC	
	*β* (95% CI)	*p*-value	*β* (95% CI)	*p*-value
Alkaline phosphatase	0.02 (0.01, 0.03)	0.005	0.07 (0.04, 0.09)	<0.001
WBC count	0.39 (0.21, 0.57)	<0.001	1.02 (0.68, 1.37)	<0.001
Neutrophil count	0.40 (0.17, 0.63)	0.001	1.08 (0.63, 1.52)	<0.001
Lymphocyte count	0.91 (0.37, 1.45)	0.002	2.06 (0.76, 3.37)	0.003

WBC, white blood cell; BMI, body mass index; WC, waist circumference; CI, confidence interval.

Model was adjusted as age, sex, education level, race, poverty, smoker, alcohol user, energy intake levels, sedentary time, total cholesterol, high-density lipoprotein cholesterol, diabetes, and hypertension.

Therefore, further mediation analysis to explore such a relationship showed that these inflammatory markers significantly mediated the associations between HbEO and BMI, and the proportions were 3.80%, 18.89%, 14.6%, and 8.86%, respectively (all *p* < 0.05). Similarly, inflammatory markers mediated the correlation between HbEO and WC at 5.70%, 21.40%, 16.80%, and 9.26% (all *p* < 0.001), respectively. Of note, the direct and indirect effects demonstrated opposite signs, which indicates that a suppression effect exists (**Table 5**).

**Table 5:** The mediation effects of inflammatory markers on the association of log2-transformed HbEO with anthropometric parameters in adults.

Outcomes	Mediators	Indirect effects	Direct effects	Total effects	Mediated proportion (%)	*P*-value
		*β* (95% CI)	*β* (95% CI)	*β* (95% CI)		
BMI	Alkaline phosphatase	0.036 (0.01, 0.07) ^*^	−0.95 (−1.13, −0.76) ^***^	0.91 (−1.10, −0.72) ^***^	3.80%	0.02
	White blood cell count	0.17 (0.10, 0.26) ^***^	−1.08 (−1.23, −0.89) ^***^	−0.91 (−1.10, −0.71) ^***^	18.89%	<0.001
	Neutrophil count	0.13 (0.06, 0.21) ^***^	−1.04 (−1.20, −0.85) ^***^	−0.91 (−1.10, −0.71) ^***^	14.6%	<0.001
	Lymphocyte count	0.08 (0.04, 0.13) ^***^	−0.99 (−1.14, −0.82) ^***^	−0.91 (−1.09, −0.73) ^***^	8.86%	<0.001
WC	Alkaline phosphatase	0.12 (0.04, 0.21) ^*^	−2.15 (−2.59, −1.63) ^***^	−2.03 (−2.51, −1.53) ^***^	5.70%	0.02
	White blood cell count	0.43 (0.26, 0.61) ^***^	−2.45 (−2.89, −1.97) ^***^	−2.03 (−2.51, −1.53) ^***^	21.40%	<0.001
	Neutrophil count	0.33 (0.17, 0.52) ^***^	−2.35 (−2.79, −1.85) ^***^	−2.02 (−2.51, −1.56) ^***^	16.80%	<0.001
	Lymphocyte count	0.18 (0.08, 0.31) ^***^	−2.21 (−2.65, −1.76) ^***^	−2.03 (−2.48, −1.54) ^***^	9.26%	<0.001

Model was adjusted as age, sex, education level, race, poverty, smoker, alcohol user, energy intake levels, sedentary time, total cholesterol, high-density lipoprotein cholesterol, diabetes, and hypertension. HbEO: hemoglobin adduct of ethylene oxide; BMI, body mass index; WC, waist circumference; CI, confidence interval; ^*^p < 0.05 and ^***^p < 0.001.

### Subgroup analysis

The results of the subgroup analyses to explore the interaction of these factors (age, sex, smoking, energy intake, sedentary time, hypertension, and diabetes) with EO-related obesity can be seen in **Tables S2** and **S3**.

Notably, the inverse association of HbEO with obesity and abdominal obesity did not differ between smokers and nonsmokers (*p* for interaction = 0.770). On the other hand, a significant inverse association remained in hypertension and diabetes subgroups across the increasing quartiles of HbEO (*p* for trend < 0.05). The comorbidity of hypertension showed a significant difference in abdominal obesity, and the comorbidity of diabetes showed a significant difference in both obesity and abdominal obesity (*p* for interaction < 0.05).

## Discussion

The present study demonstrated the association of HbEO with BMI and WC based on a US national-scale general population. We found that the level of HbEO was linearly and inversely associated with BMI and WC, which was further supported in the regression analysis for the risk of obesity. In addition, HbEO was positively associated with the inflammation markers. This relationship between HbEO and anthropometric parameters might be mediated by multiple mechanisms not expecting systemic inflammation.

Previous studies demonstrated that exposure to EO was shown to be related to inflammation (14, 26, 27). EO could cause various adverse health impacts regardless of the exposure route. Growing evidence has demonstrated that the exposure to EO was associated with the major risk factors and also the increased risk of cardiovascular diseases. Although the potential mechanism of EO-related CVD risk remained unknown, oxidative stress, insulin resistance, and inflammation might be triggered by the direct effect and intermediate product of EO (28). Also, previous studies showed that human exposure to these reactive electrophilic chemicals, such as acrylamide and EO, could contribute to human cancer or be associated with angina, heart attack, and total cardiovascular disease, and may contribute to CVD *via* inflammatory reactions and abnormal fatty acid metabolism (hypo-HDL-C and hypertriglyceride) (13, 14, 29). While both inflammation and dyslipidemia are important inducers of CVD (30–32), current results consisted of the previous studies that the increased HbEO was positively associated with various inflammatory parameters, suggesting that inflammation does contribute to this aspect.

On the other hand, obesity is a well-established risk factor for CVDs, which may also be associated with chronic systemic inflammation. Of note, WC is an indicator of visceral body fat associated with cardiometabolic disease and CVD, and a high WC may even unmask a higher CVD risk in individuals with normal weight (33, 34). Overweight and obesity recapitulate many features of the inflammatory processes (35). This obesity-related persistent inflammation could lead to endothelial dysfunction and atherosclerosis *via* the mediators released by adipose tissues (36). However, from the perspective of EO exposure and obesity, the current results firstly showed an inverse association between HbEO and risk of obesity (both overall and abdominal obesity) in adults, which were counterintuitive to a certain extent. To further explore these findings, further mediation analysis of their overlap mechanism—systemic inflammation between HbEO and anthropometric parameters—showed that suppression effects were presented in this general population, which suggested that the mechanisms of this phenomenon might extend beyond the magnitude of body weight alone.

While inverse trends in subgroups remained in these two populations, the differences in the associations between HbEO and the prevalence of abdominal obesity in hypertension and overall obesity in diabetes are consistent with these associations previously reported. Previous studies in patients with type 2 diabetes mellitus have demonstrated the phenomenon of the obesity paradox whereby higher BMI in the overweight population is associated with a lower risk of mortality (37), and patients with HTN are often associated with abdominal obesity due to the lipo-metabolism abnormalities (38, 39), which suggested that there might be a common pathway and metabolism disturbance to chronic exposure of EO, while EO exposure through smoking is the major resource in the general population and adult tobacco smokers tend to have lower BMIs and unhealthier diets relative to nonsmokers due to the increased insulin resistance, central fat accumulation, and aberrant lipolysis (1, 40, 41). Although the inverse trend remained in the smoking group, which is in line with our understanding, the current results did not differ between smokers and nonsmokers in the subgroup analysis. One explanation for the lack of a correlation in our study may be that tobacco consumption was self-reported and qualitative.

Previous studies reported that exposure to certain chemicals was associated with weight loss *via* leptin, adiponectin hormones, and the thermogenesis pathway, and may imply loss of body reserves and activation of catabolic pathways, increased catabolic cytokines leading to cardiomyocyte apoptosis and inflammation, with widespread harm (42–44). There were studies showing that the participation of EO in the pluronic modification of leptin could overcome leptin resistance at the level of the blood–brain barrier (45–47). These results indicated that chronic exposure to EO causes alterations in systemic metabolism as well as inflammation, lipolysis, and insulin resistance. In our previous study, HbEO is closely linked to serum lipid profiles (positively correlated with TC, TG, and LDL-C, and negatively correlated with HDL-C), suggesting possible dyslipidemia (15). The above mechanisms have a certain contribution to the inverse association of EO exposure derived from adduct data, which provides evidence to explore the possible mechanisms of EO-related increased CVD risk. More research is needed to determine whether the metabolic disruption and the weight changes that differ in different body components (such as gaining fat and losing muscle) are related to such CVD risk.

In summary, the exposure to EO in the general population was inversely associated with obesity and related anthropometric parameters (BMI and WC), while HbEO was positively associated with various inflammatory parameters and the mediation analysis showed a suppression effect. This implies that the EO-related CVD may not be simply explained as inflammation and dyslipidemia.

The current study has several limitations that should be considered. First, the main limitation is the cross-sectional design, and some data were self-reported by participants. Associations between exposure and parameters may change due to lifestyle factors (such as occupation), which should not be considered as direct causality and requires further validation in prospective settings. Second, as with any epidemiological analysis, our findings may be influenced by unmeasured confounding variables. Further analysis needs to consider both fat mass and dynamics of persistent organic pollutants. Data accounting for the exposure-controlled variable and related biomarkers of EO were insufficient. Lastly, multiple measurements of HbEO levels should be included in future studies to explore the cumulative effect.

## Conclusion

The current study demonstrated that higher HbEO levels were associated with an inverse trend of obesity risk and BMIs in the general adult population. Systemic inflammation may not fully account for the observed relationship between EO exposure and obesity. Further prospective and mechanistic studies are needed to validate and explore the phenomenon in the future.

## Data availability statement

The datasets presented in this study can be found in online repositories. The names of the repository/repositories and accession number(s) can be found in the article/**Supplementary Material**.

## Ethics statement

The studies involving human participants were reviewed and approved by the ethics review board of NCHS research. The patients/participants provided their written informed consent to participate in this study.

## Author contributions

IC: Conceptualization, Methodology, Writing—original draft, and Finalization. XZ: Software, Formal analysis, and Visualization. QZ: Investigation and Methodology. ML: Investigation and Methodology. SL: Data curation. ZZ: Data curation. WY: Supervision. YZ: Project administration. HZ: Supervision and Project administration. XL: Validation and Writing—reviewing and editing. All authors contributed to the article and approved the submitted version.

## Acknowledgments

We appreciate the National Center for Health Statistics (NCHS) of the Centers for Disease Control (CDC) and Prevention and all participants who enrolled in the NHANES. The author thank Dr. Yanxiu Li for the administration supports.

## Conflict of interest

The authors declare that the research was conducted in the absence of any commercial or financial relationships that could be construed as a potential conflict of interest.

## Publisher’s note

All claims expressed in this article are solely those of the authors and do not necessarily represent those of their affiliated organizations, or those of the publisher, the editors and the reviewers. Any product that may be evaluated in this article, or claim that may be made by its manufacturer, is not guaranteed or endorsed by the publisher.
