# Supplementary material for: Inverse association between blood ethylene oxide levels and obesity in the general population: NHANES 2013–2016
Source: Front Endocrinol (Lausanne). 2022 Sep 12;13:926971. doi: 10.3389/fendo.2022.926971 (PMC9510609; doi:10.3389/fendo.2022.926971)
Supplement: Supplementary file 1 [file Table_1.docx]

**Table S1 - Multiple linear regression associations of HbEO with inflammatory markers in adults.**

| Outcomes | Continuous log2-transformed HbEO | Quartile 1 | Quartile 2 | Quartile 3 | Quartile 4 | *P* for trend |
| --- | --- | --- | --- | --- | --- | --- |
|  |  | β | β (95%CI) | β (95%CI) | β (95%CI) |  |
| Alkaline phosphatase | 1.57 (0.63, 2.52) ^**^ | 0.00 (Ref.) | 1.12 (-1.28, 3.51) | 0.73 (-1.91, 3.38) | 4.24 (0.56, 7.92) ^*^ | 0.048 |
| White blood cell count | 0.34 (0.22, 0.45) ^***^ | 0.00 (Ref.) | 0.36 (0.11, 0.60) ^**^ | 0.16 (-0.12, 0.44) | 1.18 (0.79, 1.58) ^***^ | <0.001 |
| Neutrophil count | 0.25 (0.15, 0.34) ^***^ | 0.00 (Ref.) | 0.29 (0.09, 0.49) ^*^ | 0.09 (-0.13, 0.30) | 0.84 (0.53, 1.15) ^***^ | <0.001 |
| Lymphocyte count | 0.07 (0.05, 0.10) ^***^ | 0.00 (Ref.) | 0.08 (-0.02, 0.17) | 0.07 (-0.01, 0.15) | 0.28 (0.16, 0.39) ^***^ | <0.001 |

Model was adjusted for age, sex, education level, and race, poverty, smoker, alcohol user, energy intake levels, sedentary time, total cholesterol, high-density lipoprotein cholesterol, diabetes, and hypertension.

HbEO: hemoglobin adduct of ethylene oxide; CI, confidence interval; Ref., reference; ^*^ *p* < 0.05, ^**^ *p* < 0.01 and ^***^ *p* < 0.001.
