# Supplementary material for: Inverse association between blood ethylene oxide levels and obesity in the general population: NHANES 2013–2016
Source: Front Endocrinol (Lausanne). 2022 Sep 12;13:926971. doi: 10.3389/fendo.2022.926971 (PMC9510609; doi:10.3389/fendo.2022.926971)
Supplement: Supplementary file 2 [file Table_2.docx]

**Table S2 - Subgroups analysis for the associations between HbEO and the prevalence of obesity in adults.**

| Variables | Subgroups | N | Q1 | Q2 | Q3 | Q4 | *p-t* | *p-int* |
| --- | --- | --- | --- | --- | --- | --- | --- | --- |
|  |  |  | OR | OR (95%CI) | OR (95%CI) | OR (95%CI) |  |  |
| Age | <45 | 1411 | 1.00 (Ref.) | 0.63 (0.34, 1.19) | 0.41(0.23, 0.72) ^**^ | 0.34 (0.18, 0.66) ^**^ | 0.014 | 0.448 |
|  | 45-69 | 1345 | 1.00 (Ref.) | 0.68 (0.36, 1.29) | 0.79 (0.37, 1.69) | 0.45 (0.23, 0.86) ^*^ | 0.008 |  |
|  | >69 | 464 | 1.00 (Ref.) | 1.18 (0.53, 2.66) | 1.06 (0.43, 2.62) | 0.38 (0.10, 1.47) | 0.110 |  |
| Sex | Male | 1606 | 1.00 (Ref.) | 0.52 (0.28, 0.99) ^*^ | 0.47 (0.27, 0.81) ^*^ | 0.37 (0.24, 0.58) ^**^ | 0.003 | 0.890 |
|  | Female | 1614 | 1.00 (Ref.) | 0.91 (0.63, 1.33) | 0.79 (0.48, 1.28) | 0.46 (0.28, 0.75) ^**^ | 0.005 |  |
| Smoking | Yes | 1395 | 1.00 (Ref.) | 1.30 (0.58, 2.92) | 0.73 (0.38, 1.42) | 0.55 (0.32, 0.93) ^*^ | 0.003 | 0.770 |
|  | No | 1825 | 1.00 (Ref.) | 0.54 (0.37, 0.78) ^**^ | 0.59 (0.40, 0.87) ^*^ | 0.39 (0.21, 0.74) ^**^ | 0.022 |  |
| Energy intake | Low | 1073 | 1.00 (Ref.) | 0.73 (0.42, 1.24) | 0.66 (0.38, 1.13) | 0.44 (0.23, 0.84) ^*^ | 0.020 | 0.757 |
|  | Moderate | 1074 | 1.00 (Ref.) | 0.83 (0.42, 1.64) | 0.85 (0.43, 1.68) | 0.35 (0.18, 0.68) ^**^ | 0.003 |  |
|  | High | 1073 | 1.00 (Ref.) | 0.59 (0.31, 1.12) | 0.40 (0.20, 0.80) ^*^ | 0.41 (0.19, 0.86) ^*^ | 0.075 |  |
| Sedentary time | <3 hrs | 330 | 1.00 (Ref.) | 1.31 (0.57, 3.04) | 0.99 (0.43, 2.30) | 0.23 (0.07, 0.74) ^*^ | 0.005 | 0.520 |
|  | 3-6 hrs | 1424 | 1.00 (Ref.) | 0.74 (0.41, 1.33) | 0.65 (0.38, 1.09) | 0.42 (0.23, 0.76) ^*^ | 0.005 |  |
|  | >6 hrs | 1466 | 1.00 (Ref.) | 0.63 (0.40, 0.99) ^*^ | 0.56 (0.33, 0.96) ^*^ | 0.44 (0.27, 0.72) ^**^ | 0.027 |  |
| Hypertension | Yes | 1138 | 1.00 (Ref.) | 0.93 (0.51, 1.70) | 0.89 (0.49, 1.62) | 0.37 (0.21, 0.65) ^**^ | <0.001 | 0.184 |
|  | No | 2082 | 1.00 (Ref.) | 0.61 (0.38, 1.00) ^*^ | 0.48 (0.30, 0.78) ^**^ | 0.41 (0.26, 0.64) ^**^ | 0.005 |  |
| Diabetes | Yes | 425 | 1.00 (Ref.) | 0.34 (0.09, 1.36) | 0.39 (0.13, 1.17) | 0.06 (0.02, 0.23) ^***^ | <0.001 | 0.019 |
|  | No | 2795 | 1.00 (Ref.) | 0.70 (0.48, 1.04) | 0.62 (0.41, 0.93) ^*^ | 0.49 (0.35, 0.69) ^**^ | 0.003 |  |

Analyses were adjusted for covariates age, sex, education level, race, poverty, smoker, alcohol user, energy intake levels, sedentary time, total cholesterol, high-density lipoprotein cholesterol, diabetes, and hypertension when they were not the strata variables. Energy intake levels were categorized in tertiles. OR, Odd ratio; CI, confidence interval; Q: quartile; Ref., reference; *p-t*, p for trend; *p-int*, p for interaction; ^*^ *p* < 0.05, ^**^ *p* < 0.01 and ^***^ *p* < 0.001.
