# Supplementary material for: Inverse association between blood ethylene oxide levels and obesity in the general population: NHANES 2013–2016
Source: Front Endocrinol (Lausanne). 2022 Sep 12;13:926971. doi: 10.3389/fendo.2022.926971 (PMC9510609; doi:10.3389/fendo.2022.926971)
Supplement: Supplementary file 3 [file Table_3.docx]

**Table S3 - Subgroups analysis for the associations between HbEO and the prevalence of abdominal obesity in adults**

| Variables | Subgroups | N | Q1 | Q2 | Q3 | Q4 | *p-t* | *p-int* |
| --- | --- | --- | --- | --- | --- | --- | --- | --- |
|  |  |  | OR | OR (95%CI) | OR (95%CI) | OR (95%CI) |  |  |
| Age | <45 | 1411 | 1.00 (Ref.) | 0.74 (0.45, 1.24) | 0.45 (0.26, 0.77) ^**^ | 0.49 (0.26, 0.93) ^*^ | 0.127 | 0.389 |
|  | 45-69 | 1345 | 1.00 (Ref.) | 0.90 (0.41, 1.97) | 0.70 (0.31, 1.59) | 0.25 (0.10, 0.63) ^**^ | 0.003 |  |
|  | >69 | 464 | 1.00 (Ref.) | 0.79 (0.37, 1.70) | 0.52 (0.23, 1.21) | 0.39 (0.11, 1.34) | 0.149 |  |
| Sex | Male | 1606 | 1.00 (Ref.) | 0.81 (0.45, 1.47) | 0.55 (0.31, 0.98) ^*^ | 0.47 (0.28, 0.79) ^**^ | 0.013 | 0.702 |
|  | Female | 1614 | 1.00 (Ref.) | 0.87 (0.49, 1.53) | 0.55(0.27, 1.11) | 0.30 (0.13, 0.72) ^*^ | 0.009 |  |
| Smoking | Yes | 1395 | 1.00 (Ref.) | 1.24 (0.53, 2.89) | 0.81 (0.38, 1.72) | 0.54 (0.27, 1.03) | 0.009 | 0.515 |
|  | No | 1825 | 1.00 (Ref.) | 0.71 (0.45, 1.14) | 0.48 (0.29, 0.79) ^**^ | 0.46 (0.18, 1.15) | 0.081 |  |
| Energy intake | Low | 1073 | 1.00 (Ref.) | 0.58 (0.30, 1.11) | 0.40 (0.20, 0.81) ^*^ | 0.25 (0.10, 0.65) ^**^ | 0.011 | 0.696 |
|  | Moderate | 1074 | 1.00 (Ref.) | 0.90 (0.44, 1.81) | 0.78 (0.36, 1.68) | 0.46 (0.18, 1.15) | 0.077 |  |
|  | High | 1073 | 1.00 (Ref.) | 1.08 (0.57, 2.04) | 0.54 (0.29, 1.01) | 0.51 (0.27, 0.96) ^*^ | 0.052 |  |
| Sedentary time | <3 hrs | 330 | 1.00 (Ref.) | 1.49 (0.61, 3.68) | 0.69 (0.28, 1.72) | 0.31 (0.08, 1.18) | 0.052 | 0.397 |
|  | 3-6 hrs | 1424 | 1.00 (Ref.) | 0.62 (0.32, 1.18) | 0.51 (0.28, 0.92) ^*^ | 0.32 (0.17, 0.61) ^**^ | 0.003 |  |
|  | >6 hrs | 1466 | 1.00 (Ref.) | 0.95 (0.56, 1.60) | 0.57 (0.32, 0.99) ^*^ | 0.54 (0.29, 1.00) | 0.107 |  |
| Hypertension | Yes | 1138 | 1.00 (Ref.) | 1.04 (0.47, 2.30) | 0.64 (0.37, 1.10) | 0.30 (0.14, 0.65) ^**^ | 0.002 | 0.028 |
|  | No | 2082 | 1.00 (Ref.) | 0.80 (0.46, 1.39) | 0.54 (0.27, 1.05) | 0.47 (0.26, 0.86) ^*^ | 0.025 |  |
| Diabetes | Yes | 425 | 1.00 (Ref.) | 0.23 (0.03, 1.93) | 0.28 (0.05, 1.49) | 0.03 (0.00, 0.19) ^**^ | <0.001 | 0.009 |
|  | No | 2795 | 1.00 (Ref.) | 0.86 (0.55, 1.35) | 0.56 (0.33, 0.94) ^*^ | 0.46 (0.28, 0.76) ^**^ | 0.009 |  |

Analyses were adjusted for covariates age, sex, education level, race, poverty, smoker, alcohol user, energy intake levels, sedentary time, total cholesterol, high-density lipoprotein cholesterol, diabetes, and hypertension when they were not the strata variables. Energy intake levels were categorized in tertiles. OR, Odd ratio; CI, confidence interval; Q: quartile; Ref., reference; *p-t*, p for trend; *p-int*, p for interaction; ^*^ *p* < 0.05, ^**^ *p* < 0.01 and ^***^ *p* < 0.001.
